# The Role of *Phialocephala fortinii* in Improving Plants’ Phosphorus Nutrition: New Puzzle Pieces

**DOI:** 10.3390/jof8111225

**Published:** 2022-11-21

**Authors:** Vyacheslav S. Mikheev, Irina V. Struchkova, Maria N. Ageyeva, Anna A. Brilkina, Ekaterina V. Berezina

**Affiliations:** Department of Biochemistry and Biotechnology, Institute of Biology and Biomedicine, Lobachevsky State University of Nizhny Novgorod, Gagarin Avenue 23, 603950 Nizhny Novgorod, Russia

**Keywords:** dark septate endophyte, *Phialocephala fortinii*, phytase, polyphosphate, phosphorus, *Vaccinium macrocarpon*

## Abstract

Plants’ mineral nutrition in acidic soils can be facilitated by phosphate solubilizing fungi inhabiting the root systems of these plants. We attempt to find dark septate endophyte (DSE) isolates in the roots of wild-heather plants, which are capable of improving plants’ phosphorus nutrition levels. Bright-field and confocal laser scanning microscopy were used for the visualization of endophytes. A model system of co-cultivation with *Vaccinium macrocarpon* Ait. was used to study a fungal isolate’s ability to supply plants with phosphorus. Fungal phytase activity and phosphorus content in plants were estimated spectrophotometrically. In *V. vitis-idaea* L. roots, we obtained a *Phialocephala fortinii* Wang, Wilcox DSE2 isolate with acid phytase activity (maximum 6.91 ± 0.17 U on 21st day of cultivation on potato-dextrose broth medium) and the ability to accumulate polyphosphates in hyphae cells. The ability of the isolate to increase both phosphorus accumulation and biomass in *V. macrocarpon* is also shown. The data obtained for the same isolate, as puzzle pieces put together, indicate the possible mediation of *P. fortinii* DSE2 isolate in the process of phosphorus intake from inorganic soil reserves to plants.

## 1. Introduction

Phosphorus as a soil component is necessary for normal plant growth. Its deficiency in plants inhibits the formation and functioning of the most important organophosphorus compounds (nucleotides and nucleic acids, phosphates of proteins and sugars, and phospholipids) and, as a consequence, leads to a decrease in plants’ productivity because of slowing down their growth and development processes [1,2]. Since plants are important players in the global phosphorus cycle, and humanity actively consumes plants as food, raw materials, and medicines, the mechanisms and effects of phosphorus intake in plants are the constant focus of both fundamental and applied sciences [3,4].

One of the evolutionary adaptations that allow plants to use soil phosphorus reserves more effectively is their interaction with phosphate-solubilizing organisms, including filamentous fungi, soil, and endophytic and mycorrhizal species [5,6,7].

Extracellular metabolites of phosphate-solubilizing fungi (enzymes, organic acids, etc.) are able to release bound phosphate ions, acting on soil components and making them available for transport to plant roots. In addition, fungal hyphae are able to actively capture phosphate ions and store them in the form of polyphosphates or organic molecules. As a part of mycorrhiza, fungal hyphae are able to supply plants with phosphates through symbiotic interfaces in exchange for host photosynthesis products [5,8,9]. Some mycorrhizal fungi, with the help of their extra-radical hyphae, can also provide the transport of phosphate ions from soil areas outside the depletion zone around plant roots [10,11,12]. The fungi–plant interaction is of particular importance for a plant’s existence in acidic soils, in which phosphorus is included in organic (mainly in the form of phytates) or inorganic (aluminum and iron phosphates) molecules [13,14,15].

The most detailed information about the mechanisms of phosphorus exchange occurring between fungi and plants is available for glomeromycetes (Glomeromycotina) from arbuscular mycorrhizal fungi (AMF) [16,17]. However, AMF only dominate in neutral and alkaline conditions [18]. In acidic soils, their prevalence and level of plants colonization markedly decrease [19,20]. A study [21] based on meta-transcriptomic approaches clearly reveals the changes in the structure and activity of root mycobiomes in acidic soils towards the predominance of dark septate endophyte (DSE) representatives, i.e., the *Phialocephala fortinii* sensu lato (s.l.)–*Acephala applanata* species complex (PAC).

DSE fungi represent a widespread ecophysiological group of root endophytes with melanized septate hyphae and a capability of forming microsclerotia [18,22,23]. DSE in general and PAC species in particular are not host-specific fungi, and they form associations with plants from many families [24]. Together with ericoid mycorrhizal fungi, PAC species are the most common members of mycobiomes of heather plant roots. Heather plants successfully exist in harsh living conditions in acidic soils, where the plant’s access to mineral nutrition elements is difficult [15,18,25].

However, while the role of heather-specific ericoid mycorrhiza in improving phosphorus nutrition is generally recognized [8], the role of DSE in plants’ phosphorus nutrition remains partially elucidated. For some DSE isolates (strains), the positive effect on a plant’s phosphorus supply is not presented, while in others, it manifests itself in different degrees [25,26,27,28,29,30]. In our opinion, the study of various aspects of fungal effects on plants’ phosphorus nutrition values using the same isolate (strain) facilitates our understanding of the processes taking place.

We attempt to detect DSE isolates capable of improving plants’ phosphorus nutrition levels in the roots of wild-heather plants growing in the Nizhegorodsky region (Russia). This region is characterized by large soil areas with high acidity levels and an insufficient availability of phosphorus content. In order to study a number of aspects of “DSE–plant” interactions using the same DSE isolate, the following assumptions are tested:

- The roots of lingonberry (*Vaccinium vitis-idaea* L.) plants in central Russia contain DSEs belonging to the PAC;
- These endophytes are able to release phosphorus from organic compounds (phytates), as well as accumulate phosphorus in the mycelium in the form of polyphosphates;
- These endophytes are able to improve plants’ phosphorus metabolism rates and stimulate their growth during the *ex vitro* stage.

## 2. Materials and Methods

### 2.1. DSE Isolation and Identification

To isolate root endophytic fungi, wild lingonberry plants, *Vaccinium vitis-idaea* L., collected from a pine forest in central Russia, in the Nizhegorodsky region (55.7639 N, 42.5147 E), were used. Whole *V. vitis-idaea* plants were thoroughly washed with running tap water, and the roots were separated from the shoots. A total of 10 randomly selected 0.5 cm-long root segments from each of the 6 plants were superficially sterilized by immersion in 30% H_2_O_2_ solution for 60 s, and then washed 3 times in sterile, distilled water. The segments were placed in Petri dishes on potato-dextrose agar (PDA) medium and cultivated at 20 ± 2 °C in the dark for 14 days. Dark-colored, slow-growing isolates were selected. According to [30], slow-growing isolates were considered as those with a colony growth rate < 3 mm/day. The overgrown fungal mycelium was transferred to a fresh-nutrient medium and cultivated under the same conditions. The subcultivation of the isolates’ pure cultures was performed every three weeks. The colonies’ growth rate was determined during linear growth by measuring every day the colony’s diameter and calculating the average rate of change in the colony’s diameter per day. Five colonies of each isolate were analyzed.

Species identification was established in the laboratory of the All-Russian Scientific Research Institute of Agricultural Biotechnology by sequencing diagnostic loci and comparing the established sequences of DNA-ITS regions from our isolates with the reference sequences in GenBank using BLAST.

### 2.2. Microscopy of Roots and Fungi Isolates

#### 2.2.1. Bright-Field Microscopy 

Images of fungal hyphae in plant roots and the hyphae of isolated pure cultures were obtained using MT5300L/SP microscope (Meiji Techno, Saitama, Japan) equipped with a lens (Meiji Techno, Saitama, Japan) with 40× magnification and a camera (Vision Microscopy, Wiener Neudorf, Austria). To detect fungal hyphae in *V. vitis-idaea* and *V. macrocarpon* Ait. roots, root fragments were cleaned and stained according to [31]. Root fragments were treated with several portions of 10% KOH solution and heated at 85 °C for 24 h. Then, they were washed with water and stained with 0.05% trypan blue (Sigma Aldrich, St. Louis, MO, USA) solution for 1 h, and the excess dye was removed by washing with water. The stained fragments were enclosed in a 75% glycerol solution and microscoped. Some of the root fragments were analyzed without staining to detect dark-colored septate hyphae and microsclerotia.

#### 2.2.2. Confocal Laser Scanning Microscopy (CLSM) 

CLSM images of fungal isolates’ hyphae localized in *V. vitis-idaea* and *V. macrocarpon* roots were obtained using a Carl Zeiss LSM 710 confocal laser scanning system based on an Axio Observer Z1 inverted microscope (Carl Zeiss, Oberkochen, Germany), using EC Plan-Neofluar 20×/0.5 M27 or C-Apochromat 40×/1.20 W Korr M27 lenses. The preparation of samples was conducted in a manner similar to that mentioned in the previous paragraph. The fluorescence of hyphae stained with trypan blue was detected at λex = 594 nm, λem = 620–700 nm; the autofluorescence of plant root cells was detected at λex = 488 nm, λem = 499–540 nm.

To determine the localization of polyphosphates (polyPs) in fungal hyphae, CLSM images were obtained using a C-Apochromat 63×/1.20 W Korr M27 lens. To visualize polyphosphates in the hyphae, the fungal isolate was cultured for 21 days on PDA medium containing Ca_3_(PO_4_)_2_ (2 g L^–1^) as an additional component. Hyphae were placed on a slide and stained with an aqueous solution of DAPI at 50 μg/mL (Sigma Aldrich, St. Louis, MO, USA) for 30 min at room temperature, according to [32]. The fluorescence excitation of the stained polyphosphates and nuclei was conducted at λex = 405 nm, and the fluorescence signal of stained polyphosphates was received at λem = 520–609 nm, and nuclei at λem = 407–471 nm.

### 2.3. Effect of Nutrient Media on P. fortinii DSE2 Isolate Colony Growth

To determine the growth characteristics of the fungal isolate, it was cultivated in an axenic culture on 5 types of solid nutrient media: Chapek-Dox agar (CDA), CDA+Rut with the addition of rutin to a final concentration of 10 μM, CDA+Q with the addition of quercetin to a final concentration of 10 μM, and PDA and Melin–Norkrans agar (MNA). Mycelium was inoculated on fresh-nutrient medium with plugs (d = 8 mm) in the center of each Petri dish and cultivated at 20 ± 2 °C for 14 days. Colony diameter (including plug diameter) was measured in two perpendicular directions and the average of these measurements was obtained. Five colonies grown on each type of medium were analyzed.

### 2.4. Determination of the Ability of P. fortinii DSE2 Isolate to Produce Extracellular Phytases 

To identify the ability to hydrolyze organic phosphorus-containing compounds, *P. fortinii* DSE2 isolate was cultivated on potato-dextrose broth (PDB) at 20 ± 2 °C for 28 days. The isolate’s inoculation was performed by adding 3 plugs (d = 8 mm) with mycelium in 250 mL of PDB. On days 7, 14, 21, and 28, the mycelium was separated by filtration, and the filtrate was used as a crude enzyme preparation.

Phytase activity was determined according to [33]. The reaction mixture contained 500 nM of Na-phytate (Sigma Aldrich, St. Louis, MO, USA) solution, 100 mM of Na-acetate buffer (pH 4.5), and filtrate as a crude enzyme preparation. The mixture was incubated for 30 min at 37 °C. The enzyme activity was determined as the amount of released phosphate by adding the following reagent to the mixture: 10 mM ammonium molybdate solution, 5 N H_2_SO_4_ solution, and acetone at a ratio of 1:1:2. The optical density of the experimental sample was measured at λ = 355 nm on an Epoch spectrophotometer (Biotek, Santa Clara, CA, USA) against the control sample (contained buffer instead of filtrate).

Protein content in the filtrate was determined according to [34] using bovine serum albumin as a standard. The amount of phosphorus (μg) released in 1 min per 1 μg of protein was counted as 1 unit of phytase specific activity (1 U).

### 2.5. V. macrocarpon and P. fortinii DSE2 Isolate Co-Cultivation

*P. fortinii* DSE2 isolate was co-cultured with American cranberry plants (*V. macrocarpon*). *V. macrocarpon* plants were obtained from an *in vitro* culture [35] on Anderson’s nutrient medium. A total of 25 plants (the length of a single shoot was 8.2 ± 0.4 cm) with a well-developed root system were transplanted in 0.5 L pots with 0.4 L of peat, pH 4.3–4.4. At the same time, at the center of the pot, mycelium plugs (d = 8 mm) were inserted into peat to a depth of 1.5 cm, with 3 plugs per pot (inoculation variant). Fungal mycelium was obtained on PDA medium for 3 weeks. Similar plugs from PDA sterile medium were introduced into the pots with control (non-inoculated) plants. Co-cultivation was performed under lighting conditions with an intensity of 60 µmol m^−2^ s^−1^ at a photoperiod of 16 h/8 h (day/night, respectively), 22 ± 2 °C. The watering of plants was performed with a solution of mineral salts for the Anderson nutrient medium, diluted 20 times.

### 2.6. Estimation of V. macrocarpon Roots’ Mycorrhizal Colonization and Endophytic Status of P. fortinii DSE2 Isolate Confirmation

The level of *V. macrocarpon* roots’ colonization by *P. fortinii* DSE2 isolate was estimated after 5 months of co-cultivation by 3 parameters according to [36]:

1. Frequency of the DSE colonization;

F% = (number of fragments (Fs) with DSE colonization/total number of fragments);

2. Intensity of the DSE colonization in the root system

K% = (95 × D5 + 70 × D4 + 30 × D3 + 5 × D2 + D1)/(total F)

where D5 = number of fragments with colonization that rated 5; D4 = number of fragments with colonization that rated 4, etc.

3. Intensity of DSE colonization in DSE-colonized root fragments

k% = D × (total F)/(F with DSE colonization)

The endophytic status of the *P. fortinii* DSE2 isolate was confirmed by its reverse isolation from *V. macrocarpon* roots after 6 months of co-cultivation. Reverse isolation and identification were performed according to the methods described in Section 2.1. 

### 2.7. V. macrocarpon Plants’ Phosphorus Analysis

Phosphorus (P) content was determined for *V. macrocarpon* leaves and roots after 5 and 10 months of co-cultivation with *P. fortinii* DSE2 isolate. The material was dried at 85 °C to a constant weight. Leaves and roots were ground in a mortar and wet ashing was conducted in a sand bath at 370 °C for 2 h using a mixture of H_2_SO_4_: 30% H_2_O_2_ (1:0.2). The mixture was cooled and neutralized with 5 N NaOH solution in the presence of a universal indicator. Phosphorus content was determined by the phosphor-molybdenum method using ascorbic acid as a reducing agent [37]. The reaction mixture contained 0.4 mL of sample obtained after wet ashing, 2.6 mL of distilled water, 2 mL of solution containing 2.5% ammonium molybdate and 5 N H_2_SO_4_ (1:1), and 1 mL of freshly prepared 1% ascorbic acid solution. The mixture was incubated for 15 min; then, the optical density was measured on an SF-2000 spectrophotometer (OKB Spektr, St. Petersburg, Russia) at λ = 750 nm. In order to calculate the phosphorus content in the material, a calibration curve was created over the range of 25 to 100 µg phosphorus.

### 2.8. V. macrocarpon Plants’ Morphometry

After 5 and 10 months of co-cultivation of *V. macrocarpon* plants and *P. fortinii* DSE2 isolate, the fresh and dry mass of leaves, stems, and roots were determined for 10 inoculated and control plants, the number of new shoots was calculated, and their length was measured. To obtain the dry mass, drying was performed at 85 °C to a constant weight. 

### 2.9. Statistical Analysis

Statistical analysis was conducted using Microsoft Excel 2010. The difference in enzyme activity, phosphorus content in leaves and roots, number of branches, and plant biomass between inoculated and control plants were calculated using a one-way analysis of variation (ANOVA). The differences between the mean values were compared using the Mann–Whitney method with a significance level at *p* < 0.05.

## 3. Results

### 3.1. DSE in Plants: Isolation and Identification

**Figure 1:**
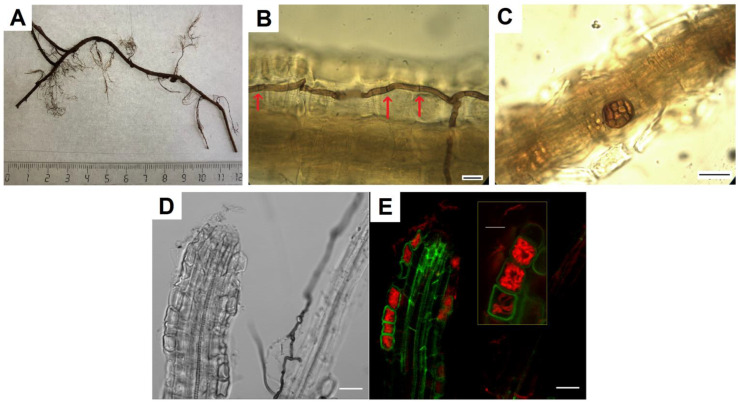
Mycobiome of *V. vitis-idaea* roots: (**A**) fragment of *V. vitis-idaea* root, from which fungi were isolated; (**B**) DSE melanized hyphae in *V. vitis-idaea* root, stained with trypan blue. Arrows indicate septa; (**C**) DSE microsclerotia in root; (**D**) transmitted light image of *V. vitis-idaea* root; (**E**) CLSM image of *V. vitis-idaea* root, stained with trypan blue. Autofluorescence of root cells (green signal) obtained at λex = 488 nm, λem = 499–540 nm; fluorescence of fungal hyphae stained with trypan blue (red signal) obtained at λex = 594 nm, λem = 620–700 nm. Bar = 10 µm.

Fungal hyphae of various types, melanized and colorless, were found in *V. vitis-idaea* roots (Figure 1A,B,D). The melanized hyphae had septa and were colored from light to dark; their thickness was about 3.5 μm. They were located on the root surface, along its central axis, and also penetrated epidermal cells and the cortex. Sometimes, dark-colored microsclerotia were observed (Figure 1C). Due to the presence of melanin in the cell walls, both types of these fungal structures were noticeable without additional staining. The use of root staining with trypan blue binding to chitin in fungal cell walls [38] made it possible to establish that, in addition to melanized hyphae, colorless hyphae were located in the root tissues, both septate and non-septate. Intracellular hyphal structures morphologically corresponding to fungal coils, typical of ericoid mycorrhiza, were identified in root epidermal cells (Figure 1C). CLSM images show that these hyphal structures are localized in root cortex cells (Figure 1D).

The isolation of slow-growing fungi obtained from 60 fragments of *V. vitis-idaea* roots using a PDA nutrient medium allowed us to obtain 57 fungal isolates. In the group of slow-growing isolates, however, differences in colony morphology and growth rate were observed, and the latter varied from 1.6 to 3 mm/day. Isolates showing similar morphologies and growth rates were grouped into seven morphotypes; isolates with a dark coloration of colonies and septated hyphae were assigned to morphotypes DSE1, DSE2, DSE3, DSE4, and DSE5 (Supplementary Table S1), while isolates with a light coloration of colonies and non-septated hyphae were assigned to Non-DSE1 and Non-DSE2.

Non-DSE1 colonies were initially white and then they turn light gray; Non-DSE2 colonies were pink. Colonies of DSE1–DSE5 morphotypes were dark, but differed in growth rate, color, and surface texture. The colors of these colonies varied from dark brown to brown-green, and colonies differed in degree of aerial mycelium development. Sporulation was not observed in any of these colonies.

According to the morphological characteristics and on the basis of comparison of their ITS sequences with sequences in GenBank, DSE1, DSE2, and DSE3 morphotypes were identified as *Phialocephala fortinii* Wang, Wilcox, whereas the DSE4 morphotype was identified as *Acephala applanata* Grünig, Sieber, i.e., all of them were PAC species. The DSE5 morphotype was identified as not belonging to PAC *P. bamuru* Wong, Dong (Supplementary Table S1).

**Figure 2:**
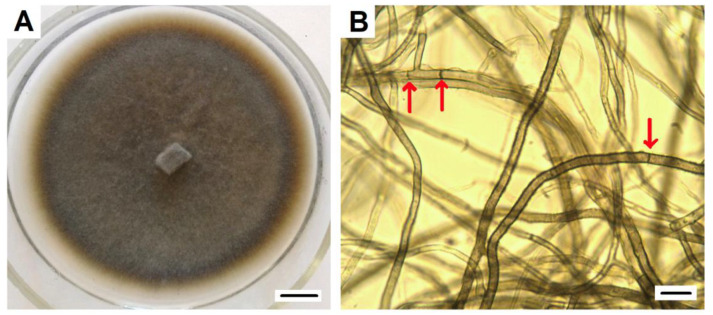
*P. fortinii* DSE2 isolate: (**A**) pure culture of *P. fortinii* DSE2 isolate. Colony developed from hyphal plug placed at the center of the Petri dish. Bar = 1 cm; (**B**) bright-field micrograph of *P. fortinii* DSE2 isolate hyphae. Arrows indicate septae. Bar = 10 µm.

The DSE2 isolate, which showed maximal similarity (100%) of ITS sequences with *P. fortinii*, including the Y1A strain isolated from *V. vitis-idaea* (sequences ID: KJ817297.1 GenBank), was used in further experiments. It was characterized by its dark-brown-colored mycelium, velvety surface with poorly developed aerial mycelium, and absence of exudate (Figure 2).

### 3.2. Effect of Nutrient Media on P. fortinii DSE2 Isolate Colony Growth

**Figure 3:**
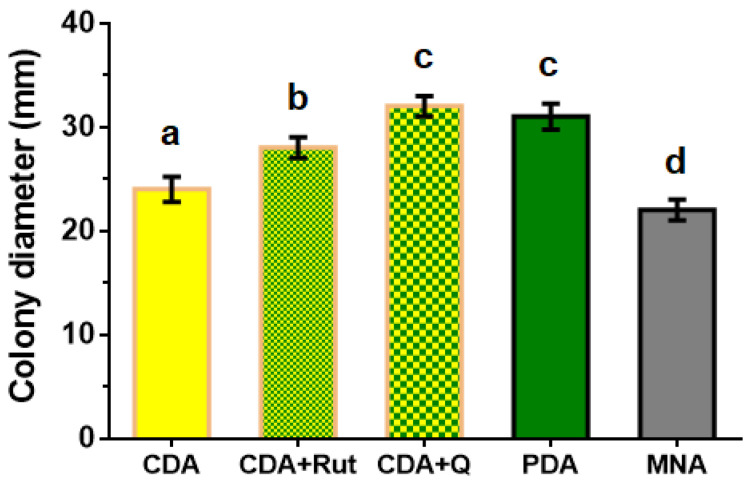
Diameter of 14-day colonies of *P. fortinii* DSE2 isolate developed on different types of nutrient media. CDA—Czapek–Dox agar medium, CDA+Rut—Czapek–Dox agar medium with the addition of rutin to final concentration of 10 μM, CDA+Q—Czapek–Dox agar medium with the addition of quercetin to final concentration of 10 μM, PDA—potato dextrose agar, and MNA—Melin–Norkrans medium. Colony diameter includes the diameter of the mycelium plug (8 mm) used for inoculation at the center of the Petri dish. Different letters indicate statistically significant differences in diameters of colonies grown on different media, *p* < 0.05.

To choose the nutrient medium for the maximal growth rate of the *P. fortinii* DSE2 isolate, it was grown on various media types. When using media containing plant components (PDA) or media with phenolic compounds characteristic of heather plants (CDA+Rut medium with the addition of rutin; CDA+Q medium with the addition of quercetin), the fungal growth rate was higher than on media consisting only of mineral salts, sucrose, and agar (CDA and MNA). On the 14th day following inoculation, colonies reached their maximum diameters on PDA and CDA+Q media (Figure 3). 

### 3.3. Measurement of P. fortinii DSE2 Isolate’s Ability to Produce Extracellular Phytases and to Accumulate Polyphosphates in Mycelium

**Figure 4:**
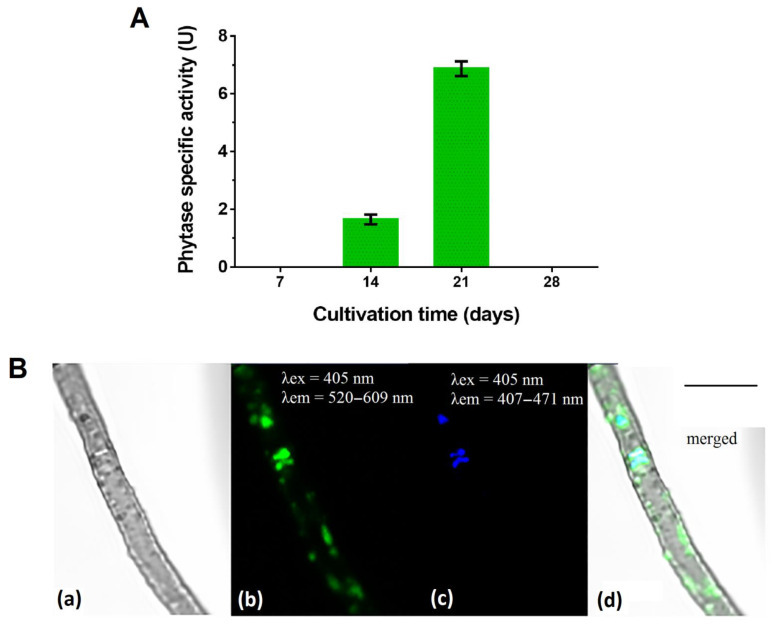
*P. fortinii* DSE2 isolate’s phytase activity and ability to accumulate phosphorus in mycelium: (**A**) *P. fortinii* DSE2 isolate’s phytase activity during cultivation on liquid potato-dextrose broth. One unit of phytase-specific activity (1 U) is counted as the amount of phosphorus (μg) released in 1 min per 1 μg of protein. Different letters indicate statistically significant differences between different days of cultivation, *p* < 0.05; (**B**) polyphosphate’s localization in *P. fortinii* DSE2-isolate hyphae stained with DAPI: (**a**) transmitted light image; (**b**) fluorescence of DAPI–polyP complexes, λex = 405 nm, λem = 520–609 nm; (**c**) fluorescence of nuclei, λex = 405 nm, λem = 407–471 nm; (**d**) merged image. Bar = 5 μm.

Phytase activity in the culture filtrate was determined for the *P. fortinii* DSE2 isolate during a 28-day growing cycle. In conditions of stationary cultivation on PDB, the specific phytase activity of this isolate was manifested on the 14th day and reached its maximum on the 21st day, when it was 6.91 ± 0.17 U (Figure 4A). In an older, 28-day-old culture, enzyme activity was absent.

We revealed that the *P. fortinii* DSE2 isolate is capable of accumulating inorganic phosphorus-containing polymer polyphosphates (polyPs) (Figure 4B). In the hyphae of the *P. fortinii* DSE2 isolate, the fluorescence of DAPI–polyP complexes was observed in the central region of the cell, where they partially overlapped with the signal from DAPI–DNA. In addition, the DAPI–polyP signal was also observed on the periphery of the cell, where the DAPI–DNA signal was absent. The fluorescence zones of DAPI–polyP on the periphery of the cells were oval and usually about 0.6 μm in size, or oblong, and 1.5–2 μm in length. The fluorescent signal of these peripheral structures was weaker than the signals of DAPI–polyP complexes in the central part of the cells.

### 3.4. Evaluation of Fungal Colonization of V. macrocarpon Roots and Confirmation of P. fortinii DSE2 Isolate’s Endophytic Status

**Figure 5:**
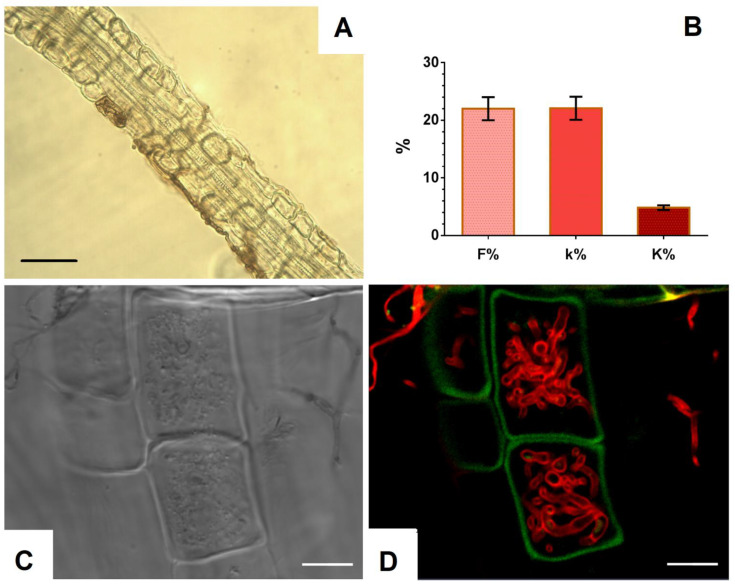
Colonization of *V. macrocarpon* roots during co-cultivation with *P. fortinii* DSE2 isolate: (**A**) melanized hyphae of *P. fortinii* DSE2 isolate in *V. macrocarpon* root after 5 months of co-cultivation. Bar = 20 µm; (**B**) indicators of colonization of *V. macrocarpon* root system after 5 months of co-cultivation: frequency of *P. fortinii* DSE2 isolate colonization (F%), intensity of *P. fortinii* DSE2 isolate colonization in DSE-colonized root fragments (k%), and intensity of *P. fortinii* DSE2 isolate colonization in the root system (K%); (**C**) transmitted light image. Bar = 30 µm; (**D**) CLSM image of *V. macrocarpon* root stained with trypan blue. Autofluorescence of root cells (green signal) obtained at λex = 488 nm, λem = 499–540 nm; fluorescence of fungal hyphae stained with trypan blue (red signal) obtained at λex = 594 nm, λem = 620–700 nm. Bar = 20 µm.

To study the ability to inhabit internal tissues of plant roots, the *P. fortinii* DSE2 isolate was co-cultivated with *V. macrocarpon* plants in pots with peat. After 5 months of co-cultivation, the hyphae of *P. fortinii* DSE2 isolate steadily colonized *V. macrocarpon* roots (Figure 5A,C,D). Bright-field microscopy (Figure 5A) revealed melanized septate hyphae in the tissues and on the surface of the roots. Using confocal microscopy, after staining with trypan blue, unmelanized hyphae were also observed in the cells and on the surface of *V. macrocarpon* roots (Figure 5C,D). Inside the cells, hyphae of various thicknesses intertwined and formed tangles. Hyphae colonization of tissues was observed in more than 20% of the root area, mainly closer to the apex in the thinnest root branches.

Reverse isolation of fungi obtained from *V. macrocarpon* roots made it possible to obtain colonies of a single morphotype corresponding to DSE2. Identification conducted by molecular genetic methods confirmed that this fungus was identical to the isolate used when co-cultivation was performed with *V. macrocarpon* (100% similarity with *P. fortinii* (KJ817297.1 GenBank)).

### 3.5. Effect of V. macrocarpon and P. fortinii DSE2 Isolate Co-Cultivation on Plants’ Phosphorus Content

**Figure 6:**
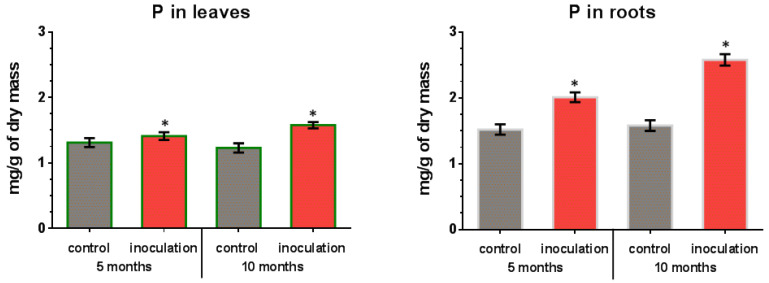
Phosphorus (P) content in *V. macrocarpon* leaves and roots after 5 and 10 months of co-cultivation with *P. fortinii* DSE2 isolate. *—phosphorus content in inoculation variant is significantly higher than in control variant, *p* < 0.05.

In leaves and roots of control (non-inoculated) plants, the phosphorus content remained almost constant throughout the experiment (Figure 6). The leaves and roots of inoculated *V. macrocarpon* plants contained a higher amount of phosphorus, which increased over time during co-cultivation. In particular, after 5 months of co-cultivation, an increase in the phosphorus content of leaves was about 8% compared to the control plants, and after 10 months it was about 28%. For the roots of inoculated plants, a more significant increase in phosphorus content was revealed: about 32% compared to control plants after 5 months, and about 61% after 10 months.

### 3.6. Effect of V. macrocarpon and P. fortinii DSE2 Isolate Co-Cultivation on Plants’ Morphological Traits

**Figure 7:**
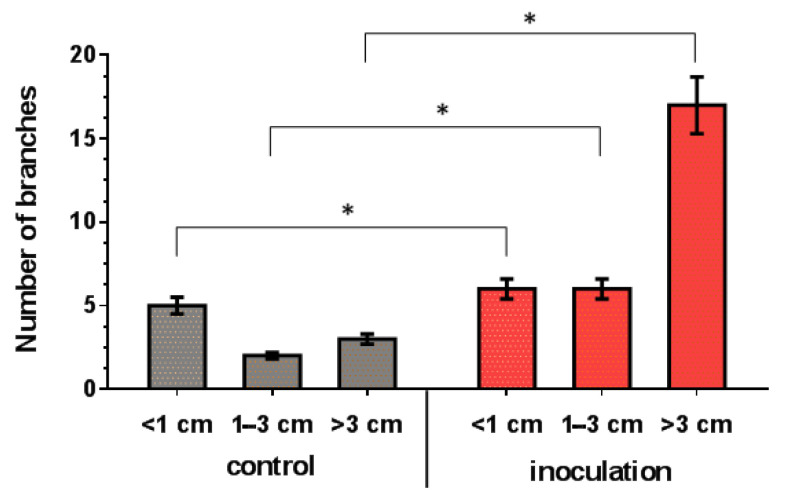
Response of length and number of *V. macrocarpon* branches to 5 months of co-cultivation with *P. fortinii* DSE2 isolate. * indicates statistically significant differences after Mann–Whitney correction was applied, *p* < 0.05.

**Figure 8:**
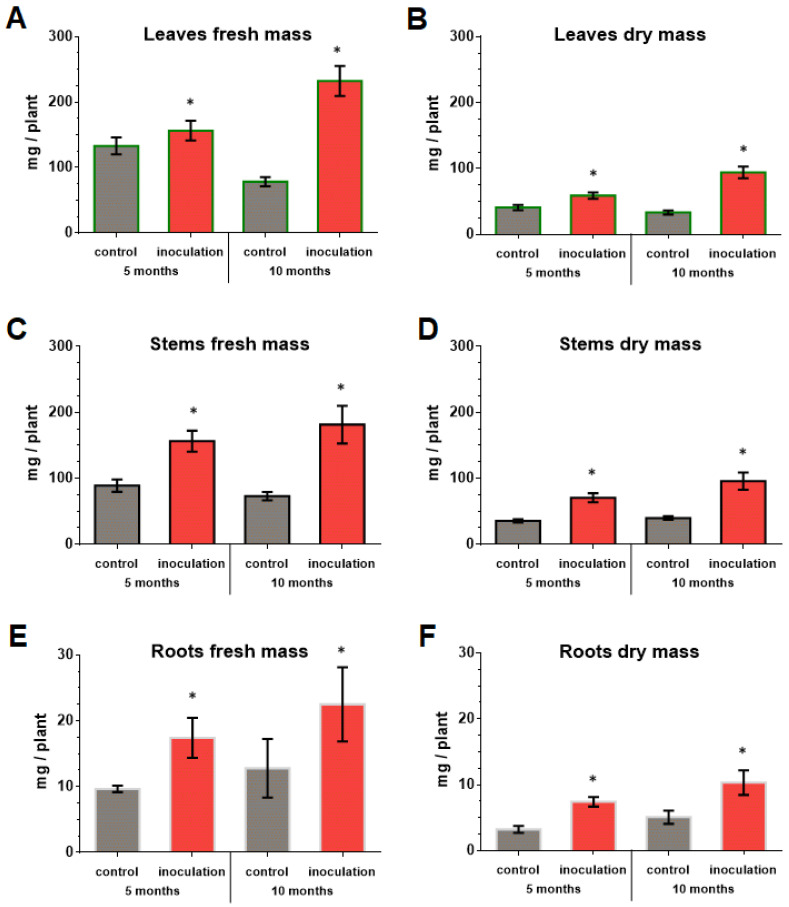
Response of *V. macrocarpon* leaves’ wet (**A**) and dry (**B**) mass, stems’ wet (**C**) and dry (**D**) mass, roots’ wet (**E**) and dry (**F**) mass after 5 and 10 months co-cultivation with *P. fortinii* DSE2 isolate. * indicates statistically significant differences after Mann–Whitney correction was applied, *p* < 0.05.

The co-cultivation of *V. macrocarpon* plants with *P. fortinii* DSE2 isolate contributed to an increase in the length and number of new shoots per plant (Figure 7), as well as the biomass of the vegetative organs of plants (Figure 8). The fresh and dry mass of plant leaves after 5 months of co-cultivation increased by 18% and 44%, fresh and dry mass of stems—by 76% and 100%, and fresh and dry mass of roots—by 81% and 130%, compared to control plants, respectively. After 10 months, the differences between control and inoculation variants were more pronounced, because the biomass of the control plants changed slightly; moreover, they even had leaf fall (Figure 8A,B).

## 4. Discussion

### 4.1. DSE in Plants: Isolation and Identification

In a new region for the DSE search (Nizhegorodsky region, Russia), we observed various morphological fungal structures in the roots of wild *V. vitis-idaea* plants, including those characteristic of DSE [39,40]: melanized hyphae running along the roots, inside or on root surface, intercellularly and intracellularly, as well as microsclerotia in root cells. Further isolation and selection of potential DSE by signs of slow growth, the dark coloration of colonies, septa in hyphae, and the absence of sporulation, allowed us to obtain isolates DSE1, DSE2, DSE3, DSE4, and DSE5, and three of them belonged to *P. fortinii* Wang, Wilcox (Ascomycota; Pezizomycotina; Leotiomycetes; Leotiomycetidae; Helotiales; Vibrisseaceae; *Phialocephala* W.B.Kendrick). Another species of PAC, *A. applanata*, is considered to be closely related to *P. fortinii* and characteristic of coniferous-tree roots [41].

For *P. fortinii*, the ability to live asymptomatically in the roots of various plants as an endophyte is well-known. The predominance of PAC and *P. fortinii* is expected for roots of the *Vaccinium* genus, since these fungi often dominate the spectra of cultivated root mycobionts of heather plants [42]. The presence of *Phialocephala* fungi in roots was revealed for heather plants in North Africa [43], China [44], the Netherlands [45], Finland [24], and Canada [46]. At the same time, species of the PAC complex are characterized by intraspecific heterogeneity with respect to their physiological and biochemical features. 

### 4.2. Effect of Culture Media on P. fortinii DSE2 Isolate Colony Growth

When comparing the growth of *P. fortinii* DSE2-isolate colonies on different types of nutrient media, we observed that fungal mycelium occupied the maximum area (the colony had a maximal diameter compared to colonies on other media types) if plant components were present, i.e., PDA, or phenolic compounds characteristic of heather plants [47,48,49], i.e., CDA with the addition of rutin or quercetin. Rapid colony growth indicates the resistance of the *P. fortinii* DSE2 isolate to concentrations of rutin and quercetin used. Moreover, it is known that flavonoids, including quercetin, are the signals between plants and fungi used in the recognition and stimulation of beneficial endophytes’ growth during arbuscular mycorrhiza [50] and ectomycorrhiza [51] formation. To date, there is no experimental evidence of a similar role of these compounds in the processes of plant interactions with DSE, but their positive effect on *P. fortinii* DSE2 isolate colony growth may indicate their function as a signal stimulating the growth of a partner fungus necessary for the host plant. The fine regulation of flavonoid synthesis in plants [52,53] makes it potentially possible for these phenolic compounds to participate in the processes of attracting *P. fortinii* to plant roots and of a symbiotic relationship’s formation with this endophyte.

### 4.3. Determination of the Ability of P. fortinii DSE2 Isolate to Produce Extracellular Phytases and Accumulate Polyphosphates in Mycelium

From 50 to 80% of soils’ organic phosphorus content is presented in the form of phytates—salts of phytic acid (myo-inositol (1,2,3,4,5,6)-hexakisphosphate). There are especially many phytates in swamp and forest soils [54]. Phytases are a special group of phosphatases that catalyze the reaction of phytate hydrolysis with phosphate release [55]. We established the presence of this enzyme’s activity in *P. fortinii* DSE2 isolate, which indicates the ability of the fungus to convert phosphates’ insoluble forms into phosphate ions, which can later be absorbed by fungi. The enzyme activity at an acidic pH (4.5) indicates its ability to function in acidic soils.

The ability of PAC fungi, namely, *P. glacialis*, *P. turiciensis*, and *A. applanata*, to hydrolyze sodium phytate was also shown in [26]. The values of enzyme activity for these species are comparable to the maximum values obtained for *P. fortinii* DSE2 isolate. At the same time, for other DSE, for example, *Exophiala pisciphila* [56], *Periconia macrospinosa*, and *Cadophora* sp. [57], the ability to secrete phytases was not detected.

For many species of fungi, it is known that phosphate ions in hyphae can be stored in the form of polyphosphates [8,17]. These polyphosphates accumulate in all organelles, mainly in vacuoles. They are used in fungal metabolism or in the process of transporting phosphorus through fungal hyphae from soil to plant as it was shown for AMF [17,58]. 

In *P. fortinii* DSE2 isolate, we observed polyphosphate inclusions on the periphery and in the center of the cell. Polyphosphates are found in vacuoles, as was shown for the *P. fortinii* strain UAMH 9608 [59]. The presence of polyphosphates has also been observed in the hyphae of other *P. fortinii* strains [30,59]. The ability to accumulate polyphosphates indicates active phosphorus metabolism and the ability to transport phosphorus into hyphae from the environment, although *P. fortinii* P-transporters are still unknown.

The ability of the *P. fortinii* DSE2 isolate to accumulate polyphosphates makes phosphorus transport through hyphae possible: from hyphae located outside the plant to hyphae growing inside it, and phosphorus transfer to the plant. Evidence of such processes has been observed in relation to AMF, indicating the proteins involved in such processes: transporters delivering phosphorus into fungal hyphae; proteins releasing phosphorus from fungus to plant; enzymes of polyphosphate turnover, etc. [8,9,60,61]. Examples of such fungal proteins are GvPT and GiPT for AMF *Glomus versiforme* and *G. intaradices*. They ensure the flow of phosphate ions released from polyphosphates in fungal hyphae into the periarbuscular space where symbiotic partners make contact.

At the same time, the *P. fortinii* DSE2 isolate may create polyphosphate reserves for its own needs and may not have special mechanisms for their transfer to the plant. For DSE as a whole, the absence of a specialized symbiotic interface is usually noted. However, even in this case, fungi cytoplasmic content movement to growing hyphae tips [62] can cause the spatial closure of phosphorus from the soil to plant and its release when hyphae die near or inside the plant.

### 4.4. Effect of V. macrocarpon and P. fortinii DSE2 Isolate Co-Cultivation on Plants’ Growth and Phosphorus Content

For a number of *P. fortinii* strains isolated in various regions, during their co-cultivation with plants, an increase in phosphorus accumulation by host plants, including heather plants, was recorded [63].

To study the effect of *P. fortinii* DSE2 isolate on the possibility of supplying plants with phosphorus, a model system of its co-cultivation with *V. macrocarpon* was used. The possibility of the artificial colonization of plants with DSE fungi isolated from other plant species has been shown earlier [28].

In our study, the ability of *P. fortinii* DSE2 isolate to increase both phosphorus and biomass accumulation values in *V. macrocarpon* was demonstrated. The ability of various fungi, from yeast [17] to mycelial ascomycetes of the DSE group [56], to transport phosphorus into plant tissues was noted. Though plant-phosphorus accumulation largely depends on fungal strain [25], the meta-analysis of data related to the effects of plants co-cultivation with *P. fortinii* showed an increased accumulation of phosphorus in tissues of various plants [64].

We showed that co-cultivation with *P. fortinii* DSE2 isolate increased the biomass of all parts of *V. macrocarpon* plants; however, information about other representatives of the PAC complex is contradictory. A number of researchers have shown that plant co-cultivation with representatives of the *Phialocephala* genus reduces or does not affect plant biomass accumulation [65,66]. This effect can vary from strain to strain, for example, it was shown [25] that different strains of *P. fortinii* increased the biomass accumulation of rhododendrons to various degrees.

Phosphorus-intake increase in plants enhances the growth of the plant on the whole, and root development and stem thickening in particular. Phosphorus is a component of structural and regulatory molecules; it activates and deactivates enzymes as part of macroergic compounds. Since most of the organic phosphate is incorporated into nucleic acids, plant phosphorus intake stimulates nucleic acids and protein biosynthesis, cell division, and, as a result, biomass accumulation [67]. The availability of phosphorus affects the expression of a number of proteins: carbohydrate-transporting proteins, photosystem-component proteins, RUBISCO, chlorophyll-synthesis enzymes, etc. [68]. Phosphorus is also involved in many other metabolic processes, for example, in photosynthesis, which is a process that ensures biomass accumulation by fixing carbon into carbohydrates [67]. In particular, phosphorus provides the energization of thylakoids, positively affecting CO_2_ assimilation by increasing the ATP-dependent regeneration of ribulose-1,5-bisphosphate in the Calvin cycle [68]. 

Thus, the data obtained in our study not only indicate the beneficial effect of *P. fortinii* DSE2 isolate on the biomass accumulation of *V. macrocarpon* plants, but also indicate that this fungus forms a symbiotic relationship with the plant. This is also evidenced by the fact that after 10 months of co-cultivation, the plant biomass continued to increase, which means that the favorable symbiotic relationship of the fungus with the plant is able to persist for a long time. 

## 5. Conclusions

The role of DSE participation in plants’ phosphorus nutrition as well as underlying mechanisms of such a participation is a serious question, and an answer, similar to a puzzle, is assembled from fragments, many pieces of which are missing at present. 

In our study, *P. fortinii* DSE2 isolate was found in the roots of *V. vitis-idaea* plants in the territory of central Russia, a new DSE search region. This isolate was able to colonize the roots of plants of another species, *V. macrocarpon*, to increase the plant’s phosphorus content and biomass, and showed the ability to hydrolyze phytates, as well as accumulate polyphosphates. We assumed that these puzzle pieces may signify the mediation of *P. fortinii* DSE2 isolate in the process of phosphorus intake from inorganic soil reserves by plants. In the future, this assumption and alternative hypotheses about the improvement of plants’ phosphorus intake due to *P. fortinii* saprotrophic activity, which does not require its endophytic existence, will be tested, as well as the hypothesis about the indirect participation of fungi in improving plants’ phosphorus nutrition levels through changes in plant physiology and morphology with the help of signaling compounds of interspecies communication. The use of the DSE isolate would open up further possibilities for investigating its interactions with other wild and cultivated plants, which would undoubtedly help to better understand the role of this fungi group in plant phosphorus nutrition studies. 

## Data Availability

Data are contained within the article and Supplementary Materials.

## Supplementary Materials

The following supporting information can be downloaded at: https://www.mdpi.com/article/10.3390/jof8111225/s1, Table S1: characteristics of isolates—representatives of slow-growing dark septate endophyte morphotypes from *V. vitis-idaea* roots (DSE1–5 isolates).

## References

[1] Rodriguez D., Zubillaga M.M., Ploschuk E.L., Keltjens W.G., Goudriaan J., Lavado R.S. (1998). Leaf area expansion and assimilate production in sunflower (*Helianthus annuus* L.) growing under low phosphorus conditions. Plant Soil.

[2] Kavanová M., Lattanzi F.A., Grimoldi A.A., Schnyder H. (2006). Phosphorus Deficiency Decreases Cell Division and Elongation in Grass Leaves. Plant Physiol..

[3] Bailey-Serres J., Parker J.E., Ainsworth E.A., Oldroyd G.E.D., Schroeder J.I. (2019). Genetic strategies for improving crop yields. Nature.

[4] Bindraban P.S., Dimkpa C.O., Pandey R. (2020). Exploring phosphorus fertilizers and fertilization strategies for improved human and environmental health. Biol. Fertil. Soils.

[5] Alori E.T., Glick B.R., Babalola O.O. (2017). Microbial Phosphorus Solubilization and Its Potential for Use in Sustainable Agriculture. Front. Microbiol..

[6] Dandessa C. (2020). Review on phosphate solubilizing fungi and its inoculation to seeds. Int. J. Curr. Res. Acad. Rev..

[7] Andrino A., Guggenberger G., Kernchen S., Mikutta R., Sauheitl L., Boy J. (2021). Production of Organic Acids by Arbuscular Mycorrhizal Fungi and Their Contribution in the Mobilization of Phosphorus Bound to Iron Oxides. Front. Plant Sci..

[8] Plassard C., Becquer A., Garcia K. (2019). Phosphorus Transport in Mycorrhiza: How Far Are We?. Trends Plant Sci..

[9] Nguyen C.T., Saito K. (2021). Role of Cell Wall Polyphosphates in Phosphorus Transfer at the Arbuscular Interface in Mycorrhizas. Front. Plant Sci..

[10] Jakobsen I., Joner E.J., Larsen J., Gianinazzi S., Schüepp H. (1994). Hyphal phosphorus transport, a keystone to mycorrhizal enhancement of plant growth. Impact of Arbuscular Mycorrhizas on Sustainable Agriculture and Natural Ecosystems.

[11] Plassard C., Louche J., Ali M.A., Duchemin M., Legname E., Cloutier-Hurteau B. (2011). Diversity in phosphorus mobilisation and uptake in ectomycorrhizal fungi. Ann. For. Sci..

[12] Ezawa T., Saito K. (2018). How do arbuscular mycorrhizal fungi handle phosphate? New insight into fine-tuning of phosphate metabolism. New Phytol..

[13] Vohník M., Albrechtova J. (2011). The Co-occurrence and Morphological Continuum Between Ericoid Mycorrhiza and Dark Septate Endophytes in Roots of Six European *Rhododendron* Species. Folia Geobot..

[14] Stroheker S., Dubach V., Queloz V., Sieber T.N. (2018). Resilience of *Phialocephala fortinii* s.l.—*Acephala applanata* communities—Effects of disturbance and strain introduction. Fungal Ecol..

[15] Landolt M., Stroheker S., Queloz V., Gall A., Sieber T.N. (2020). Does water availability influence the abundance of species of the *Phialocephala fortinii* s.l.—*Acephala applanata* complex (PAC) in roots of pubescent oak (*Quercus pubescens*) and Scots pine (*Pinus sylvestris*)?. Fungal Ecol..

[16] Smith F.W. (2002). The phosphate uptake mechanism. Plant Soil.

[17] Bhalla K., Qu X., Kretschmer M., Kronstad J.W. (2022). The phosphate language of fungi. Trends Microbiol..

[18] Malicka M., Magurno F., Piotrowska-Seget Z. (2022). Plant association with dark septate endophytes: When the going gets tough (and stressful), the tough fungi get going. Chemosphere.

[19] Wang G.M., Stribley D.P., Tinker P.B., Walker C. (1993). Effects of pH on arbuscular mycorrhiza I. Field observations on the long-term liming experiments at Rothamsted and Woburn. New Phytol..

[20] Helgason T., Fitter A.H. (2009). Natural selection and the evolutionary ecology of the arbuscular mycorrhizal fungi (Phylum *Glomeromycota*). J. Exp. Bot..

[21] Young E., Carey M., Meharg A.A., Meharg C. (2018). Microbiome and ecotypic adaption of *Holcus lanatus* (L.) to extremes of its soil pH range, investigated through transcriptome sequencing. Microbiome.

[22] Rodriguez R.J., White J.F., Arnold A.E., Redman R.S. (2009). Fungal endophytes: Diversity and functional roles. New Phytol..

[23] Stroheker S., Dubach V., Vögtli I., Sieber T. (2021). Investigating Host Preference of Root Endophytes of Three European Tree Species, with a Focus on Members of the *Phialocephala fortinii*—*Acephala applanata* Species Complex (PAC). J. Fungi.

[24] Jumpponen A., Trappe J.M. (1998). Dark septate endophytes: A review of facultative biotrophic root-colonizing fungi. New Phytol..

[25] Vohník M., Albrechtová J., Vosátka M. (2005). The inoculation with *Oidiodendron maius* and *Phialocephala fortinii* alters phosphorus and nitrogen uptake, foliar C: N ratio and root biomass distribution in *Rhododendron* cv. Azurro. Symbiosis.

[26] Della Monica I.F., Saparrat M.C.N., Godeas A.M., Scervino J.M. (2015). The co-existence between DSE and AMF symbionts affects plant P pools through P mineralization and solubilization processes. Fungal Ecol..

[27] Barrow J.R., Osuna P. (2002). Phosphorus solubilization and uptake by dark septate fungi in fourwing saltbush, *Atriplex canescens* (Pursh) Nutt. J. Arid Environ..

[28] Surono, Narisawa K. (2018). The inhibitory role of dark septate endophytic fungus *Phialocephala fortinii* against *Fusarium* disease on the Asparagus officinalis growth in organic source conditions. Biol. Control.

[29] Surono, Narisawa K. (2017). The dark septate endophytic fungus *Phialocephala fortinii* is a potential decomposer of soil organic compounds and a promoter of *Asparagus officinalis* growth. Fungal Ecol..

[30] Yu T., Nassuth A., Peterson R.L. (2001). Characterization of the interaction between the dark septate fungus *Phialocephala fortinii* and *Asparagus officinalis* roots. Can. J. Microbiol..

[31] Massicotte H.B., Melville L.H., Peterson R.L. (2005). Structural characteristics of root–fungal interactions for five ericaceous species in eastern Canada. Can. J. Bot..

[32] Gomes F.M., Ramos I., Wendt C., Girard-Dias W., De Souza W., Machado E.A., Miranda E.A.K. (2013). New insights into the in situ microscopic visualization and quantification of inorganic polyphosphate stores by 4′,6-diamidino-2-phenylindole (DAPI)-staining. Eur. J. Histochem..

[33] Greiner R., Haller E., Konietzny U., Jany K.-D. (1997). Purification and Characterization of a Phytase from *Klebsiella terrigena*. Arch. Biochem. Biophys..

[34] Lowry O.H., Rosebrough N.J., Farr A.L., Randall R.J. (1951). Protein measurement with the Folin phenol reagent. J. Biol. Chem..

[35] Berezina E.V., Brilkina A.A., Veselov A.P. (2017). Content of phenolic compounds, ascorbic acid, and photosynthetic pigments in *Vaccinium macrocarpon* Ait. dependent on seasonal plant development stages and age (the example of introduction in Russia). Sci. Hortic..

[36] Trouvelot A., Kough J.L., Gianinazzi-Pearson V., Gianinazzi-Pearson V., Gianinazzi S. (1986). Mesure du taux de mycorhization VA d’un systeme radiculaire. Recherche de methodes d’estimation ayant une signification fonctionelle. Physiological and Genetical Aspects of Mycorrhizae.

[37] Lowry O.H., Lopez J.A. (1946). The determination of inorganic phosphate in the presence of labile phosphate esters. J. Biol. Chem..

[38] Liesche J., Marek M., Gã¼Nther-Pomorski T. (2015). Cell wall staining with Trypan blue enables quantitative analysis of morphological changes in yeast cells. Front. Microbiol..

[39] Sieber T.N., Waisel Y., Eshel A., Kafkafi I. (2002). Fungal root endophytes. Plant Roots: The Hidden Half.

[40] Sietiö O.-M., Tuomivirta T., Santalahti M., Kiheri H., Timonen S., Sun H., Fritze H., Heinonsalo J. (2018). Ericoid plant species and *Pinus sylvestris* shape fungal communities in their roots and surrounding soil. New Phytol..

[41] Grünig C.R., Sieber T.N. (2005). Molecular and phenotypic description of the widespread root symbiont *Acephala applanata* gen. et sp. nov., formerly known as dark-septate endophyte Type 1. Mycologia.

[42] Vohník M. (2020). Ericoid mycorrhizal symbiosis: Theoretical background and methods for its comprehensive investigation. Mycorrhiza.

[43] Hamim A., Miché L., Douaik A., Mrabet R., Ouhammou A., Duponnois R., Hafidi M. (2017). Diversity of fungal assemblages in roots of *Ericaceae* in two Mediterranean contrasting ecosystems. Comptes Rendus. Biol..

[44] Tanney J.B., Seifert K.A. (2020). Mollisiaceae: An overlooked lineage of diverse endophytes. Stud. Mycol..

[45] Zijlstra J.D., Van’t Hof P., Baar J., Verkley G.J.M., Summerbell R.C., Paradi I., Braakhekke W.G., Berendse F. (2005). Diversity of symbiotic root endophytes of the *Helotiales* in ericaceous plants and the grass, *Deschampsia flexuosa*. Stud. Mycol..

[46] Addy H.D., Hambletonb S., Currah R.S. (2000). Distribution and molecular characterization of the root endophyte *Phialocephala fortinii* along an environmental gradient in the boreal forest of Alberta. Mycol. Res..

[47] Venskutonis P.R., Barnackas Š., Kazernavičiūtė R., Maždžierienė R., Pukalskas A., Šipailienė A., Labokas J., Ložienė K., Abrutienė G. (2016). Variations in antioxidant capacity and phenolics in leaf extracts isolated by different polarity solvents from seven blueberry (*Vaccinium* L.) genotypes at three physiological stages. Acta Physiol. Plant..

[48] Bujor O.-C., Ginies C., Popa V.I., Dufour C. (2018). Phenolic compounds and antioxidant activities of lingonberry (*Vaccinium vitis-idaea* L.) leaf, stem and fruit at different harvest periods. Food Chem..

[49] Jurikova T., Skrovankova S., Mlcek J., Balla S., Snopek L. (2018). Bioactive Compounds, Antioxidant Activity, and Biological activity of European Cranberry (*Vaccinium oxycoccos*). Molecules.

[50] Bécard G., Douds D.D., Pfeffer P.E. (1992). Extensive In Vitro Hyphal Growth of Vesicular-Arbuscular Mycorrhizal Fungi in the Presence of CO_2_ and Flavonols. Appl. Environ. Microbiol..

[51] Gomes B., Castro F., Santos R., Figueiredo P., Silva M., Vidal M., Ferreira I., Nunes J., Machado H., Gomes F. (2021). Effect of Quercetin on Mycorrhizal Synthesis between *Tuberborchii* and *Arbutusunedo* L. In Vitro Plants. Microbiol. Res..

[52] Larose G., Chênevert R., Moutoglis P., Gagné S., Piché Y., Vierheilig H. (2002). Flavonoid levels in roots of *Medicago sativa* are modulated by the developmental stage of the symbiosis and the root colonizing arbuscular mycorrhizal fungus. J. Plant Physiol..

[53] Lillo C., Lea U.S., Ruoff P. (2008). Nutrient depletion as a key factor for manipulating gene expression and product formation in different branches of the flavonoid pathway. Plant Cell Environ..

[54] Liu X., Han R., Cao Y., Turner B.L., Ma L.Q. (2022). Enhancing Phytate Availability in Soils and Phytate-P Acquisition by Plants: A Review. Environ. Sci. Technol..

[55] Wyss M., Brugger R., Kronenberger A., Rémy R., Fimbel R., Oesterhelt G., Lehmann M., Van Loon A.P.G.M. (1999). Biochemical characterization of fungal phytases (myo-inositol hexakisphosphate phosphohydrolases): Catalytic properties. Appl. Environ. Microb..

[56] Xu R., Li T., Shen M., Yang Z.L., Zhao Z.-W. (2020). Evidence for a Dark Septate Endophyte (*Exophiala Pisciphila*, H93) Enhancing Phosphorus Absorption by Maize Seedlings. Plant Soil.

[57] Yakti W., Kovács G.M., Vági P., Franken P. (2018). Impact of dark septate endophytes on tomato growth and nutrient uptake. Plant Ecol. Divers..

[58] Werner T.P., Amrhein N., Freimoser F.M. (2007). Specific localization of inorganic polyphosphate (poly P) in fungal cell walls by selective extraction and immunohistochemistry. Fungal Genet. Biol..

[59] Saito K., Kuga-Uetake Y., Saito M., Peterson R.L. (2006). Vacuolar localization of phosphorus in hyphae of *Phialocephala fortinii*, a dark septate fungal root endophyte. Can. J. Microbiol..

[60] Ezawa T., Cavagnaro T., Smith S.E., Smith F.A., Ohtomo R. (2004). Rapid accumulation of polyphosphate in extraradical hyphae of an arbuscular mycorrhizal fungus as revealed by histochemistry and a polyphosphate kinase/luciferase system. New Phytol..

[61] Ferrol N., Azcón-Aguilar C., Pérez-Tienda J. (2019). Arbuscular mycorrhizas as key players in sustainable plant phosphorus acquisition: An overview on the mechanisms involved. Plant Sci..

[62] Klein D.A., Paschke M. (2004). Filamentous Fungi: The Indeterminate Lifestyle and Microbial Ecology. Microb. Ecol..

[63] Vohník M., Lukančič S., Bahor E., Regvar M., Vosátka M., Vodnik D. (2003). Inoculation of *Rhododendron* cv. Belle-Heller with two strains of *Phialocephala fortinii* in two different substrates. Folia Geobot..

[64] Newsham K.K. (2011). A meta-analysis of plant responses to dark septate root endophytes. New Phytol..

[65] Tellenbach C., Grünig C.R., Sieber T.N. (2011). Negative effects on survival and performance of Norway spruce seedlings colonized by dark septate root endophytes are primarily isolate-dependent. Environ. Microbiol..

[66] Mayerhofer M.S., Kernaghan G., Harper K. (2013). The effects of fungal root endophytes on plant growth: A meta-analysis. Mycorrhiza.

[67] Malhotra H., Sharma S., Pandey R., Hasanuzzaman M., Fujita M., Oku H., Nahar K., Hawrylak-Nowak B. (2018). Phosphorus nutrition: Plant growth in response to deficiency and excess. Plant Nutrients and Abiotic Stress Tolerance.

[68] Hammond J.P., White P. (2008). Sucrose transport in the phloem: Integrating root responses to phosphorus starvation. J. Exp. Bot..

