# Supplementary material for: The Role of Phialocephala fortinii in Improving Plants’ Phosphorus Nutrition: New Puzzle Pieces
Source: J Fungi (Basel). 2022 Nov 21;8(11):1225. doi: 10.3390/jof8111225 (PMC9695368; doi:10.3390/jof8111225)
Supplement: Supplementary file 1 [file jof-08-01225-s001.zip › jof-2047277-supplementary.pdf]

Supplementary Table S1. Characteristics of isolates – representatives of slow-growing dark septate endophytes morphotypes from *V. vitis-idaea* roots (DSE1–5 isolates)

Isolate – morphotype representative	Average growth rate, cm/day	Colony appearance	Accession number	Accordance %
DSE1	0.3 ± 0.02	Olive-brown 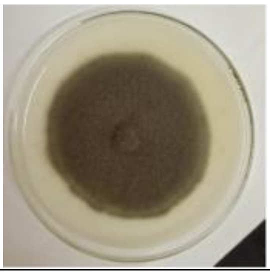	*Phialocephala fortinii* KJ817297.1	99.64
DSE2	0.24 ± 0.01	Dark brown 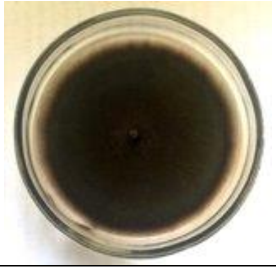	*Phialocephala fortinii:* MT276008.1, MH931279.1, LC131022.1, etc.	100
DSE3	0.24 ± 0.03	Dark brown Abundant areal mycelium 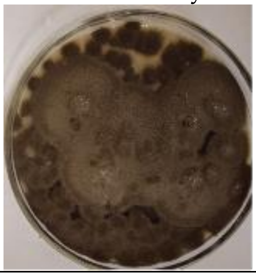	*Phialocephala bamuru* MG195534.1	99.64
DSE4	0.24 ± 0.01	Dark brown 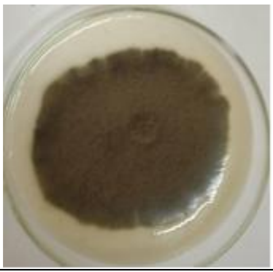	*Phialocephala fortinii* MT276008.1	99.82
DSE5	0.16 ± 0.01	Dark brown 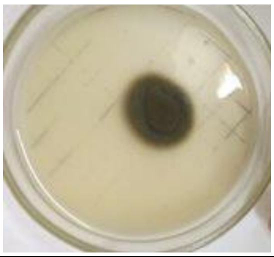	*Acephala* sp. HQ889709.1	98.51
